# The neural determinants of age-related changes in fluid intelligence: a pre-registered, longitudinal analysis in UK Biobank

**DOI:** 10.12688/wellcomeopenres.14241.2

**Published:** 2018-06-15

**Authors:** Rogier A. Kievit, Delia Fuhrmann, Gesa Sophia Borgeest, Ivan L. Simpson-Kent, Richard N. A. Henson

**Affiliations:** 1 MRC Cognition and Brain Sciences Unit, University of Cambridge, Cambridge, Cambridgeshire , CB2 7EF, UK

**Keywords:** Aging, cognitive aging, fluid intelligence, Biobank, white matter, grey matter, individual differences, structural equation modelling

## Abstract

**Background: **Fluid intelligence declines with advancing age, starting in early adulthood. Within-subject declines in fluid intelligence are highly correlated with contemporaneous declines in the ability to live and function independently. To support healthy aging, the mechanisms underlying these declines need to be better understood.

**Methods: **In this pre-registered analysis, we applied latent growth curve modelling to investigate the neural determinants of longitudinal changes in fluid intelligence across three time points in 185,317 individuals (N=9,719 two waves, N=870 three waves) from the UK Biobank (age range: 39-73 years).

**Results: **We found a weak but significant effect of cross-sectional age on the mean fluid intelligence score, such that older individuals scored slightly lower. However, the mean longitudinal slope was positive, rather than negative, suggesting improvement across testing occasions. Despite the considerable sample size, the slope variance was non-significant, suggesting no reliable individual differences in change over time. This null-result is likely due to the nature of the cognitive test used. In a subset of individuals, we found that white matter microstructure (N=8839, as indexed by fractional anisotropy) and grey-matter volume (N=9931) in pre-defined regions-of-interest accounted for complementary and unique variance in mean fluid intelligence scores. The strongest effects were such that higher grey matter volume in the frontal pole and greater white matter microstructure in the posterior thalamic radiations were associated with higher fluid intelligence scores.

**Conclusions: **In a large preregistered analysis, we demonstrate a weak but significant negative association between age and fluid intelligence. However, we did not observe plausible longitudinal patterns, instead observing a weak increase across testing occasions, and no significant individual differences in rates of change, likely due to the suboptimal task design. Finally, we find support for our preregistered expectation that white- and grey matter make separate contributions to individual differences in fluid intelligence beyond age.

## Introduction

Fluid intelligence refers to the ability to solve novel problems in the absence of task-specific knowledge, and predicts important outcomes including life expectancy, expected income and work performance ( Gottfredson & Deary, 2004). Both cross-sectional (e.g. Hartshorne & Germine, 2015; Kievit *et al.*, 2016) and longitudinal studies (e.g. Ghisletta *et al.*, 2012; Salthouse, 2009; Schaie, 1994) have shown that advancing age is associated with a marked decrease in fluid intelligence performance. Although the precise starting point of decline is hard to estimate precisely due to cohort effects, selective attrition and enrolment and retest effects in longitudinal cohorts (e.g. Salthouse *et al.*, 2004), estimates for the onset of decline in fluid intelligence range between the third (e.g. Park *et al.*, 2002; Salthouse, 2009) and sixth decade of life (e.g. Schaie, 1994). Moreover, recent findings have demonstrated that within-subject decline in fluid intelligence is highly correlated with within-subject declines in the ability to live and function independently ( Tucker-Drob, 2011). The advent of large-scale neuroimaging studies has shown that neural measures can be strongly predictive of individual differences in fluid intelligence (e.g. Kievit *et al.*, 2014; Ritchie *et al.*, 2015). A better understanding of the neural determinants of changes in fluid intelligence is therefore necessary for improving our understanding of healthy cognitive aging, and may aid the development of early markers for individuals at risk of rapid decline. Recent innovations in multivariate models allow researchers to simultaneously estimate multiple determinants of current ability as well as changes in ability over time ( Jacobucci *et al.*, 2018). To estimate these models with precision, large datasets are required. The UK Biobank ( Sudlow *et al.*, 2015) is a unique resource for addressing such questions, as it includes both cognitive and neural measures on an unprecedented number of participants.

In our pre-registration, we proposed analyses of UK Biobank cognitive and brain data to a) examine the nature of age-related decline in fluid intelligence and b) model the neural determinants of this decline. The cognitive data consisted of the Biobank’s fluid intelligence scores, which were acquired in N=185,317 people (aged 39–73 years) across up to three testing occasions 2–4 years apart (though note that the majority of individuals only completed one (174,728) or two (9,719) assessments). The brain data came from a subset of approximately 10,000 individuals (white matter data, grey matter data) who underwent an MRI scan, and consisted of pre-processed measures of the integrity of major white-matter tracts (N=8839) and volume of grey matter (N=9931) in key brain regions ( Miller *et al.*, 2016). Our preregistered analyses entailed two steps: first modelling cognitive data; second including neuroimaging predictors of cognitive abilities. More specifically, our pre-registered analyses specified the use of latent growth models ( Bauer, 2007) to model the mean and slope of age-related changes in fluid intelligence, in order to address the following questions:

1. What is the magnitude of change in fluid intelligence across occasions, as captured by the slope of fluid intelligence?
2. Is there significant variance associated with this slope (i.e. do people differ in their rate of change)?
3. Is the slope linear or non-linear (i.e. does a quadratic latent growth factor capture meaningful variance above a linear factor)?
4. Does the rate of decline (slope) depend on the level (intercept) (i.e. is age-related decline determined by current cognitive status)?
5. Is there evidence for subgroups (growth mixture models) (i.e. do we find evidence of subgroups of individuals, differing in their baseline score or rate of change)?

On the basis of prior studies, we predicted a decline in fluid intelligence across testing occasions. We expected that the decline in fluid intelligence would be more pronounced in older individuals ( Kievit *et al.*, 2014), and that there would be significant individual differences in the rate of change ( Ghisletta *et al.*, 2012). We had no strong expectations about slope-intercept covariance or the presence of subgroups.

Our second set of hypotheses concerned the neural determinants of individual differences in the slope and intercept of fluid intelligence. To examine this question, we preregistered a series of analyses using Multiple Indicator Multiple Causes (MIMIC) models ( Jöreskog & Goldberger, 1975; Kievit *et al.*, 2012) to relate the mean and slope estimates for fluid intelligence to the various brain measures, and asked:

6. What neural properties determine the intercept and slope of fluid intelligence?
7. Are the neural determinants of the mean (general ability) the same as those of the slope (rate of change)?
8. Do multiple region-specific markers of neural health predict unique variance in cognitive level and slope, or does a single global marker suffice?

Based on prior work, we predicted that the mean and/or slope estimates from the latent growth models will depend in particular on complementary effects of frontal grey and white matter ( Kievit *et al.*, 2014; Kievit *et al.*, 2016). Moreover, we expected the slope and intercept to have similar, but non-identical multiple brain determinants, as the mechanisms that govern individual differences need not be identical to those governing within-subject change (cf. Kievit *et al.*, 2013). We also pre-registered exploratory analyses relating possible sub-groups to factors like physical health, but given the insufficient evidence for sub-groups, we did not explore these relationships further.

## Methods

### Participants

The present study sample consisted of a subset of healthy middle to older-aged adults (age range at time of recruitment: 39–73 years) from the UK Biobank cohort (for more information see the Biobank website; Sudlow *et al.*, 2015). Participants were recruited between 2006 and 2010 via the UK National Health Service. UK Biobank received ethical approval from the North West Multi-Centre Research Ethics Committee (11/NW/03820). Although a total of 502,655 participants took part in Biobank, we focus on 185,317 individuals who have data for at least one wave of fluid intelligence testing. Testing took place at 22 assessment centres across the UK with each participant completing lifestyle, demographic, health and mood questionnaires, cognitive assessments and physical measures (e.g. blood, saliva and urine samples). We here analysed fluid intelligence and neurological data downloaded in 2017. Fluid ability was measured up to three times for each participant, with intervals of approximately 2–4 years (M±SD t2-t1: 4.29±1.01 years; M±SD t3-t2: 2.56±0.84 years). There were 165,491, 20,042 and 9,167 participants at waves 1, 2, and 3, respectively (note that a subject could have their first assessment in the second wave). Despite sizeable attrition, the current dataset provides in principle sufficient power to detect any non-trivial effect(s) and enables sensitive model comparisons ( Hertzog *et al.*, 2008). All analyses reported below can be reproduced or modified using scripts made available in the supplementary materials, namely Kievit_etal_biobank_dataprep.R (data preparation; Supplementary File 1); Kievit_etal_biobank_analysis.R (analyses and plots; Supplementary File 2); Kievitetal_GFGMM1.inp (growth mixture models in Mplus; Supplementary File 3). To acquire the raw data, one can register and apply through the central biobank portal.

### Fluid ability measures

**Figure 1.**
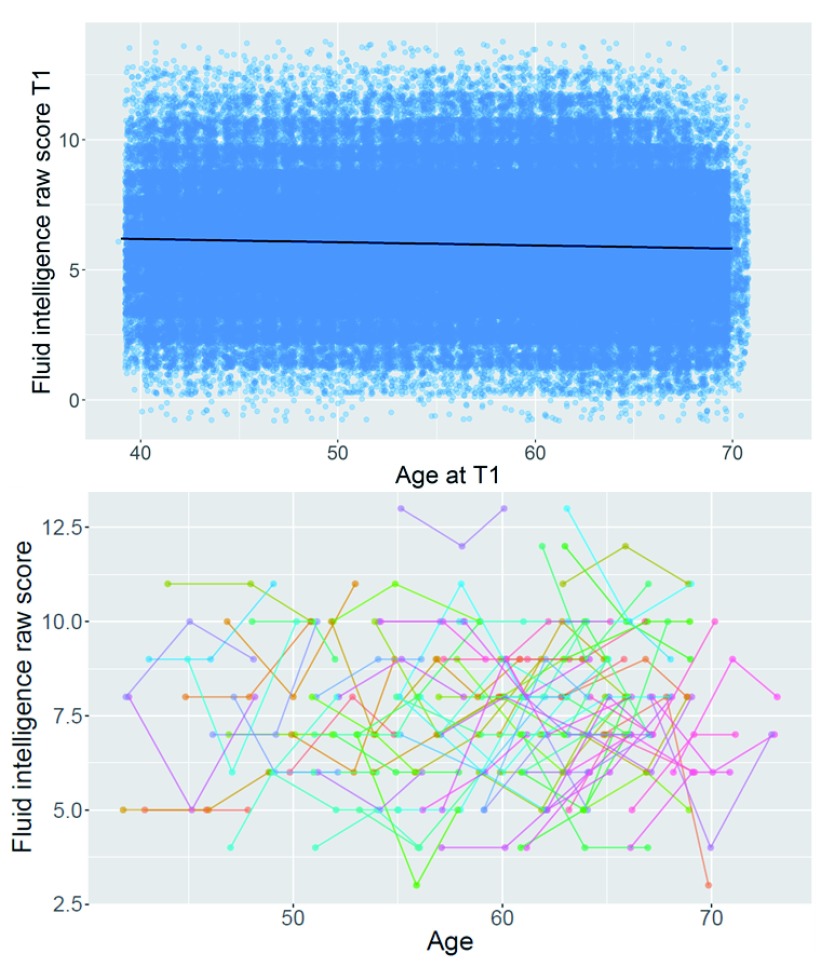
Top: Linear relation between age and fluid intelligence sumscores at Time 1 (some jitter added for visibility). Bottom: A random subsample of raw fluid intelligence scores across testing occasions.

We here analysed the ‘fluid intelligence test’ included in the UK Biobank cognitive battery. The test is designed to measure “the capacity to solve problems that require logic and reasoning ability, independent of acquired knowledge” (for a complete overview of the 13 individual fluid intelligence items, please see the Biobank manual for the Fluid intelligence test). The test comprised thirteen logic and reasoning questions administered via a computer-touchscreen interface with a two-minute time limit for each question. The maximum score was 13 (one point for each correct response). Overall, the test items have a reported Cronbach alpha coefficient of 0.62 ( Hagenaars *et al.*, 2016). No participants or observations were excluded from subsequent analyses. Raw data are shown for the fluid intelligence scores at T1 ( Figure 1, top), and a random subset of 100 individuals with 3 timepoints ( Figure 1, bottom).

**Figure 2.**
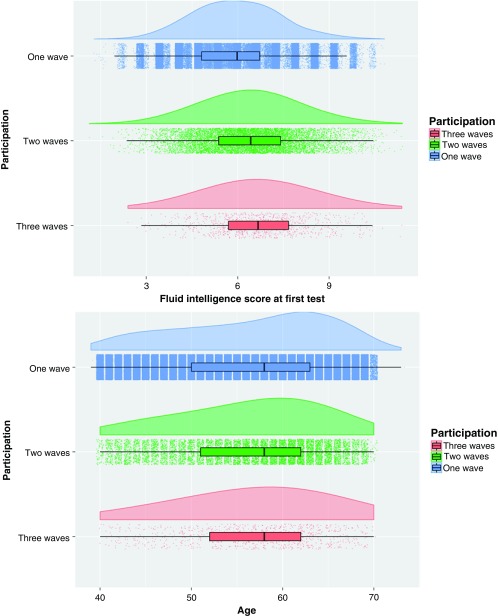
Intelligence intercept scores (top) and age at first testing occasion (bottom) as a function of the number of measurement occasions (one, two or three). Individuals who took part in all three waves were slightly older, and scored slightly higher on the fluid intelligence task.

Participants who took part in all three waves (N=870) were slightly older, and had lightly higher baseline scores, than those who took part in only one or two waves (See Figure 2, top and bottom). By using all available data, under the assumption of Missing At Random (i.e. the attrition is associated with variables also included in the model) using Full Information Maximum likelihood should yield unbiased estimates (cf. Enders & Bandalos, 2001)

### Neural measures: grey and white matter components

In order to assess how individual differences in the microstructure of major white matter tracts contribute to fluid ability, we used a mean tract-based estimate of fractional anisotropy (FA) (see Miller *et al.*, 2016, for more details on the Biobank imaging pipeline). We chose FA because previous studies of white matter in healthy aging have mostly used FA, and because FA has been shown to be a comparatively reliable metric ( Fox *et al.*, 2012; for nuances regarding the interpretation of FA, see Jones *et al.*, 2013 or Wandell, 2016). Note that Biobank also includes various other white matter metric of interest including diffusivity (MD), Neurite Orientation and Dispersion and others – These measures have specific strengths and weaknesses (see Cox *et al.*, 2016, for a discussion of the merits of more novel metrics) that are beyond the remit of this manuscript. We started with 27 tracts ( Miller *et al.*, 2016), and averaged bilateral hemispheric tracts, yielding mean FA estimates for a total of 15 tracts: acoustic radiation, anterior thalamic radiation, cingulate gyrus, parahippocampal part of cingulum, corticospinal tract, forceps major, forceps minor, inferior fronto-occipital fasciculus, inferior longitudinal fasciculus, middle cerebellar peduncle, medial lemniscus, posterior thalamic radiation, superior longitudinal fasciculus, superior thalamic radiation and uncinate fasciculus. Quality control was conducted by both automated identification of e.g. outlier slices and SNR, as well as manual inspection – For more detail, see Miller *et al.*, 2016, online methods.

For grey matter, we selected grey matter regions based on the Parieto-Frontal Integration Theory P-FIT ( Jung & Haier, 2007). P-FIT postulates a network of cortical brain regions as the brain substrate of intelligence. The proposed network includes the dorsolateral prefrontal cortex, the inferior and superior parietal lobules, the anterior cingulate gyrus and selected areas within the temporal and occipital lobes. Recent studies have offered support for P-FIT ( Hoffman *et al.*, 2017; Ryman *et al.*, 2016). Following our pre-registered specification to include 10 GM regions, we selected the following 10 ROIs, bilaterally averaged: the frontal pole, superior frontal gyrus, middle frontal gyrus, inferior frontal gyrus (pars triangularis and pars opercularis subdivision), supramarginal gyrus (posterior and anterior), angular gyrus, frontal medial cortex and the cingulate gyrus.

### Structural equation modelling (SEM)

Models were estimated using the Lavaan version 0.5-23.1097 ( Rosseel, 2012) package for SEM in R version 3.4.2 (Short summer) ( R Development Core Team, 2016). We used the full information maximum likelihood estimator (FIML) to use all available data and the robust maximum likelihood estimator with a Yuan-Bentler scaled test statistic (MLR) to account for violations of multivariate normality. We further assessed overall model fit via the Satorra-Bentler scaled test statistic along with the chi-square test, the root mean square error of approximation (RMSEA) with its confidence interval, the Comparative Fit Index (CFI), and the standardized root mean squared residuals (SRMR) ( Schermelleh-Engel *et al.*, 2003). Using these indices, good fit was defined as: RMSEA (acceptable fit < 0.08, good fit < 0.05), CFI (acceptable fit 0.95 - 0.97, good fit > 0.97), SRMR (acceptable fit 0.05 - 0.10, good fit < 0.05).

## Results

### Fluid intelligence latent growth curve model

To test our pre-registered behavioural analyses, we used a latent growth curve model (LGCM), as shown in Figure 2. We fit the model to the full sample (N=185,317) with three time points, using FIML estimation to account for missingness. The slope factor loadings were constrained to the mean intervals between timepoint 1 and 2 (4.3) and 1 and 3 (6.85). This model fit the data well: χ ^2^(2) = 10.70, p = 0.005; RMSEA = 0.005 [0.002 - 0.008]; CFI = 0.999; SRMR = 0.006. Raw parameter estimates are shown in Figure 2. The mean score at T1 was 6.706, with a strong suggestion of individual differences (intercept variance estimate=2.955, SE=0.116, z=25.39, with a significant decrease in model fit when constraining the intercept variance: χ2(1), 549.6, p<0.0001). Higher age was associated with slightly lower intercepts (estimate= -0.013, SE= 0.001, z=-19.809, see also Figure 1A). However, this effect was very small (standardized path=-0.06), especially compared to previously reported effects (e.g. r=-0.7, Kievit *et al.*, 2014). The pattern of results for the slopes was unexpected. First, the slope intercept (in this specification the mean change per measurement occasion) was strongly positive (estimate=0.208, SE=0.017, z=12.602), suggesting people, on average, improved over time. In other words, there was no evidence of our hypothesized within-subject age-related cognitive decline. There was a weak negative effect of age on slope (est=-0.002, SE=0.0001, z=-7.018) suggesting older individuals improved slightly less than younger adults. Most surprisingly, the slope variance was non-significant and *negative* (est=-0.001, SE=0.004) suggesting an improper solution. Suggesting an improper solution. A likelihood ratio test showed the slope variance could be constrained to 0 without adversely affecting model fit χ2(1), .63, p=.72). This indicates that there were no reliable indications of individual differences in change over time. Although non-significant slope variance has been reported previously for fluid intelligence over time ( Yuan *et al.*, 2018), and improper solutions are common in random effects models ( Eager & Roy, 2017), it is nonetheless highly surprising in a sample of this magnitude. To achieve a proper solution we therefore constrained the slope variance and slope-intercept covariance to 0 (for this and future models), and refit the model, which yielded good model fit χ ^2^(4) = 10.88, p = 0.028; RMSEA = 0.003 [0.001 – 0.005]; CFI = 0.999; SRMR = 0.006) and showed negligible changes to other parameter estimates compared to the model without constraints (final parameters shown in Figure 2). In line with our preregistered analysis 1c, we also fit a quadratic growth model by including a quadratic growth factor with linear factor loadings squared, and imposed constraints in order to render the model identifiable (residual variances equality constrained across occasions, and linear slope variance constrained to 0 based on the linear model). However, this model too yielded an improper solution (a negative quadratic slope variance), so it cannot be interpreted with confidence.

To further examine the unexpected absence of a negative slope or reliable slope variance, we examined a set of alternative, exploratory, analytic approaches and model specifications. First, in the previous analysis we used full information maximum likelihood to analyze all individuals, despite considerable missing data. Comparable results were obtained when fitting the same models to reduced subsets of the data (e.g. only those with at least two (9,719), or all three measurements (N=870). We attempted to address two further plausible explanations for the poor quality of the longitudinal data. Firstly, we fit a second-order latent growth curve model, where fluid intelligence was measured by 13 observed indicators at every time point, imposing equal factor loadings across occasions. Such a model could appropriately weigh individual items based on the degree to which they share variance, possibly improving the purity of the fluid intelligence estimates. Although this model yielded a significant slope variance ^1^, other aspects of model fit were poor, including factor loadings (mean standardized factor loading for T1=0.14), and model indices such as the CFI (0.133) and SRMR (0.150) suggested poor fit. As substantive patterns were similar to the occasion sum scores (i.e. positive slope intercept) we will continue with the first order growth model instead. In a final exploratory analysis, we reran the basic growth model with every individual item. This yielded qualitatively very similar results, with positive slopes for all items and non-significant slopes for all but one item (item 5). Closer inspection of item 5 suggested only a marginal, uncorrected benefit of freely estimating the slope variance χ2(1), 8.1, p=.004, combined with a non-significant slope intercept, and a BIC favouring the constrained slope model, together suggesting insufficient evidence to proceed with this post hoc item selection instead of the sumscore.

One likely explanation for the increase across testing occasions is the presence of practice effects (e.g. Salthouse, 2010). To address this explanation, we fit another exploratory model including an additional growth factor with factor loadings constrained to 0, 1 and 1 for the three time points. This so-called ‘boost’ factor (Hoffman *et al.*, 2012) captures the hypothesis that test performance will show an improvement between the first and second testing occasions that is purely a practice effect. The inclusion of the boost factor rendered the slope intercept non-significant, which is compatible with the notion that the gains are most likely practice gains. However, like the quadratic model, such a more complex model is only identified by imposing a range of constraints (here including constraining the boost factor variance to 0). Moreover, despite these constraints this model yielded an improper solution and should thus be interpreted with caution. In a final exploratory analysis, we switched from an occasion-specific approach (T1, T2, T3) to an age-specific approach (scores at a given age). Although this approach yielded high proportions of missing data (as every individual will have missing data for most ages), it has been successfully applied to study cognitive aging ( Ghisletta & Lindenberger, 2003) and can allow for more convenient decomposition of retest effects. However, this approach too failed to converge. In summary, we conclude that a meaningful longitudinal signal does not exist in the repeated measures fluid intelligence task, as currently implemented in Biobank.

**Figure 3.**
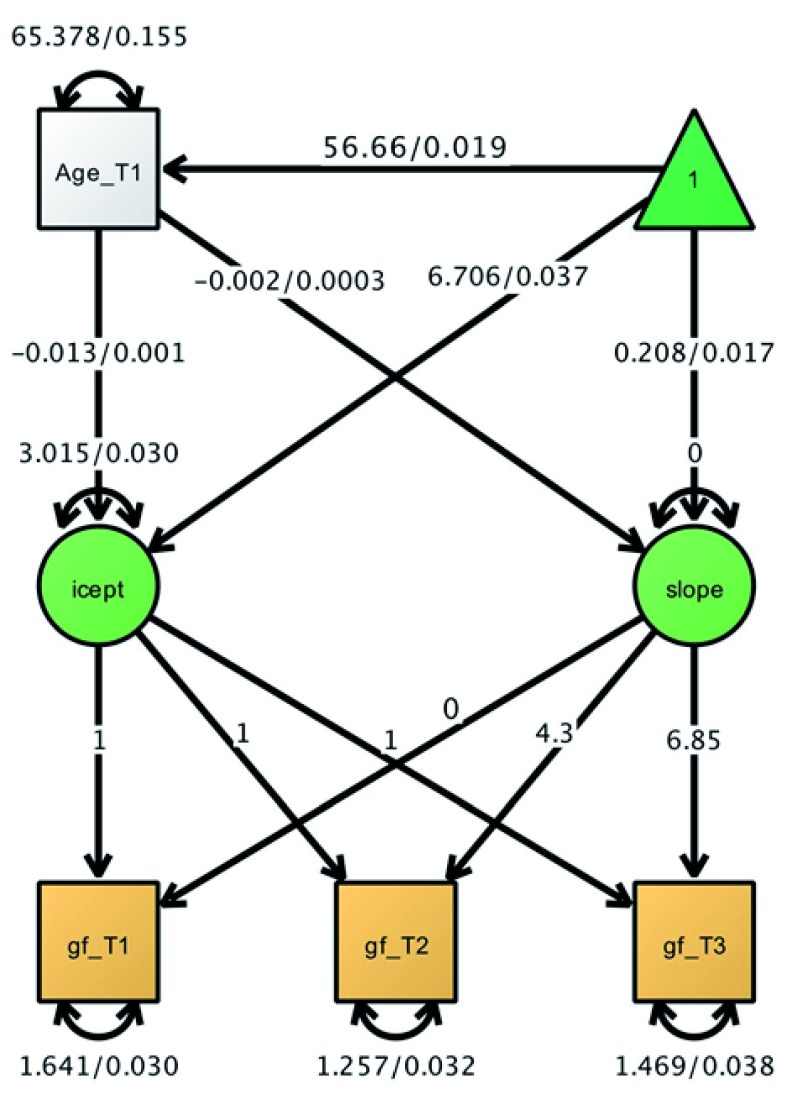
Latent growth curve model for fluid intelligence sum scores across 3 occasions. Plot shows beta/standard errors. gf = fluid intelligence. T=timepoint. icept= intercept.

Finally, in line with our preregistered analyses (1e), we fit a series of growth mixture models to examine evidence for the presence of subgroups. For this analysis, we used Mplus (version 7.4 ( Muthén & Muthén, 2005). We fit 1 to 5 classes and examined the sample size adjusted BIC (SA-BIC) to decide on the best model. As shown in Figure 3, the SA-BIC was lowest for the four-group solution. However, further inspection of this solution suggested that evidence for subgroups was weak. Firstly, the ‘best’ solution of 4 subgroups had poor entropy (0.61, Figure 3 right panel), well below common guidelines of 0.8. This suggests subgroups were not well separated. More importantly, inspection of the slopes and intercepts showed that the four subgroups were effectively subdividing the normal distribution of the whole population into subgroups (i.e. two larger groups with an intercept/slope close to the population mean, two smaller groups with intercept/slopes closer to the upper and lower ‘edges’ of the population distribution). This pattern of results is common in growth mixture modelling ( Bauer, 2007, p. 768, Figure 3). Therefore, we conclude that there is no compelling evidence for latent subgroups with different longitudinal patterns. We now turn to our examination of the neural determinants of fluid intelligence.

### White matter determinants of fluid intelligence

Next, in line with our second set of preregistered analyses, we fit a LGM-MIMIC model, where both the intercept and slope were regressed simultaneously on neural predictors. First, we focus on white matter. We started by testing our preregistered prediction whether the scores across tracts can be reduced to a single factor, which would suggest that a single global factor suffices (preregistration 2c), or whether individual ROIs are required. We observed that a model with a single white matter latent variable measured by all 15 tracts fit poorly (χ ^2^(90) = 8023.57, p < 0.001; RMSEA = 0.100 [0.099 - 0.101]; CFI = 0.957; SRMR = 0.061), replicating previous findings ( Kievit *et al.*, 2016; Lövdén *et al.*, 2013), and suggesting further analyses should include individual tracts. In all further models, age was included as a covariate of both intercept and slope, estimation was conducted on the full sample using FIML, and all tracts were allowed to co-vary with each other, as well as with age (not shown in figures for visual clarity).

**Figure 4.**
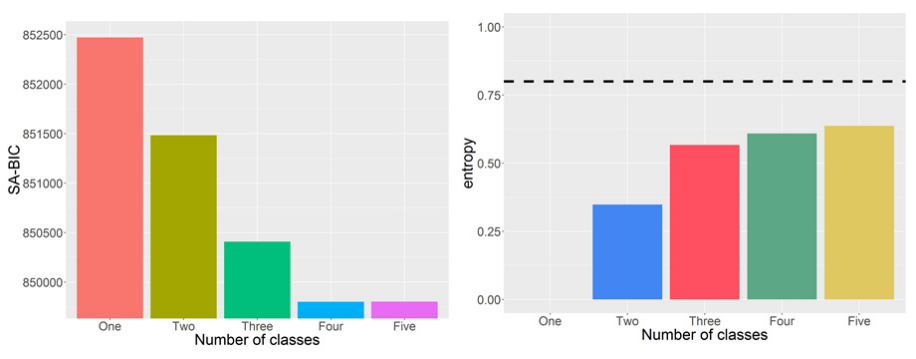
Sample size adjusted Bayesian Information Criterion (BIC), left, and entropy (right) for 1–5 classes in a growth mixture model approach. Dashed line indicates commonly accepted entropy criterion for good separation.

First, the full model LGM-MIMIC model fit the data well (χ ^2^(19) = 19.06, p = 0.453; RMSEA = 0.0001 [.000 - 0.002]; CFI = 1.000; SRMR = 0.004. In this model, the intercept of fluid ability was significantly associated with FA in five tracts, as shown in Figure 4. Jointly the tracts and age explained 2.1% of the variance in fluid intelligence, equivalent to a standardized effect of r=0.145, which is small by individual differences standards ( Gignac & Szodorai, 2016). Higher FA predicted higher fluid ability in all significant tracts apart from the forceps major and the inferior fronto-occipital fasciculus. Contrary to our expectation and previous findings, the forceps minor was not the strongest predictor of the fluid intelligence intercept ( Kievit *et al.*, 2014, Figure 4). None of the white tracts predicted slope variance - A likelihood ratio test showed that the regression paths of the slope on the individual tracts could be constrained to 0 without adversely affecting model fit χ2(15), 17.97, p=.26. Next, we examined grey matter volume correlates of the fluid intelligence intercept.

### Grey matter determinants of fluid intelligence

**Figure 5.**
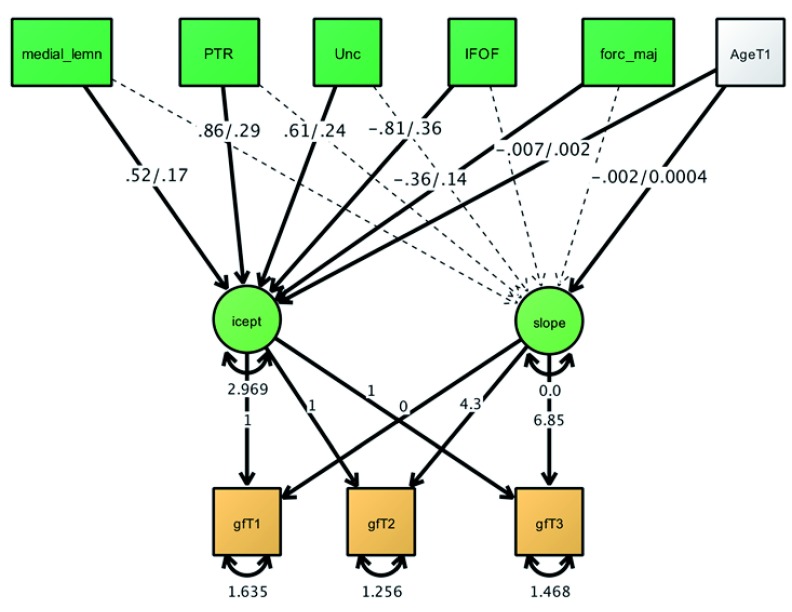
Multiple Indicator, Multiple Causes (MIMIC) model of fluid intelligence and white matter tracts showing 5 significant predictors, jointly predicting 1.3% of the variance in gf. Plot shows beta/standard errors. Non-significant tracts and tract covariances were estimated but are omitted for clarity. gF= fluid intelligence. icept= intercept. medial_lemn: medial lemniscus; PTR: posterior thalamic radiation; Unc: uncinate fasciculus; IFOF: inferior fronto-occipital fasciculus; forc_maj: forceps major.

Next, we fit the same model using only estimates of grey matter volume. First, we again replicated the poor fit of a single factor model, suggesting that a global grey matter factor does not accurately reflect the population covariance structure (χ ^2^(35) = 7208.61, p < 0.001; RMSEA = 0.144 [0.141 - 0.146]; CFI = 0.783; SRMR = 0.071). Next, we estimated a joint LGM MIMIC model as above, which showed good model fit (χ ^2^(14) = 15.01, p = 0.377; RMSEA = 0.001 [0.0 - 0.002]; CFI = 1.000; SRMR = 0.003). The joint effect size of 4.5% was considerably larger than for white matter (albeit still modest). Inspection of key parameters (see Figure 5) showed that the strongest determinant of the fluid intelligence intercept was the frontal pole (r=.16), replicating our previous finding in a separate cohort ( Kievit *et al.*, 2014, Figure 4). Two additional regions, namely the angular gyrus and the inferior frontal gyrus, explained further variance in the fluid intelligence intercept. No regions predicted slope variance - A likelihood ratio test showed the regression paths of the slope on the individual regions could be constrained to 0 without adversely affecting model fit χ2(10), 12.55, p=.24.

### Joint Grey matter and white matter determinants of fluid intelligence

**Figure 6.**
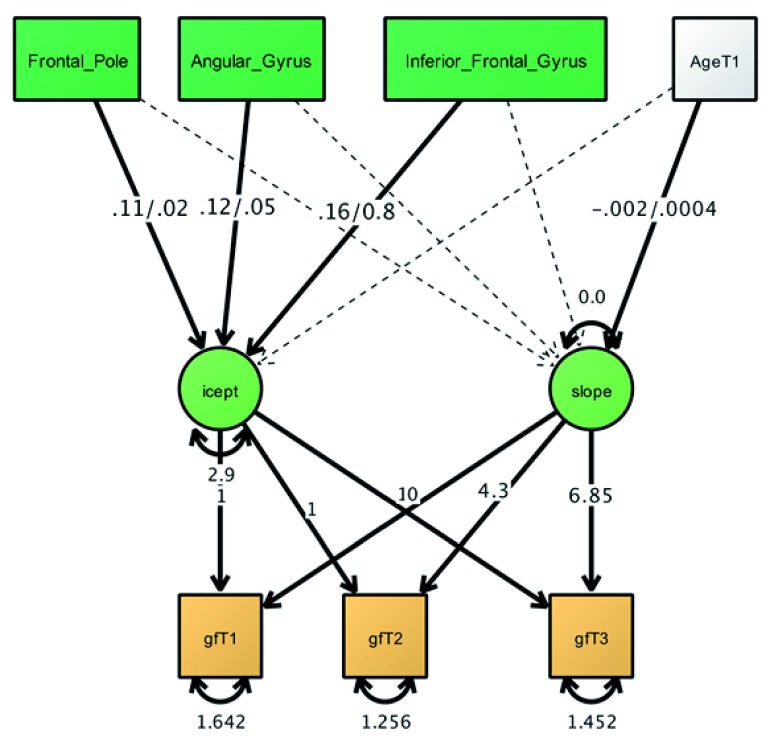
Multiple Indicator, Multiple Causes (MIMIC) Latent Growth (LGM) model for fluid intelligence and grey matter, jointly predicting 4.5% of the variance. All paths shown are beta/standard errors. Non-significant tracts and tract covariances were estimated but are omitted for clarity.

Finally, we examined whether the grey and white matter provide complementary information about fluid intelligence, in line with our preregistered prediction. To do so, we refit the above MIMIC model, including only those white and grey matter regions that were nominally significant in the modality-specific analyses. Again, model fit was good (χ ^2^(14) = 16.11, p = 0.186; RMSEA = 0.001 [.0000000 - 0.003]; CFI = 1.000; SRMR = 0.004), with a joint effect size of 5.2% (intercept) variance explained. Inspection of the parameter estimates supported our *a priori* hypothesis regarding the intercept: grey matter volume and white matter microstructure made largely complementary contributions to individual differences in fluid intelligence. The two strongest paths were (again) grey matter in the frontal pole (r=0.16) and white matter in the posterior thalamic radiations (r=0.12). Together, these findings support our preregistered hypotheses that white matter and grey matter would provide partly complementary effects. As before, no regions or tracts predicted slope variance, χ2(10), 10.99, p=.35. As there was no meaningful slope variance, we could not address our preregistered expectation that neural determinants would be similar but distinct for intercept and slope. Contrary to our *a priori* hypothesis, frontal white matter was not the strongest determinant of individual differences in fluid intelligence. Instead, in the full model, the posterior thalamic radiations, a posterior tract linking the occipital lobe to the thalamus, proved most strongly predictive ( Figure 6).

**Figure 7.**
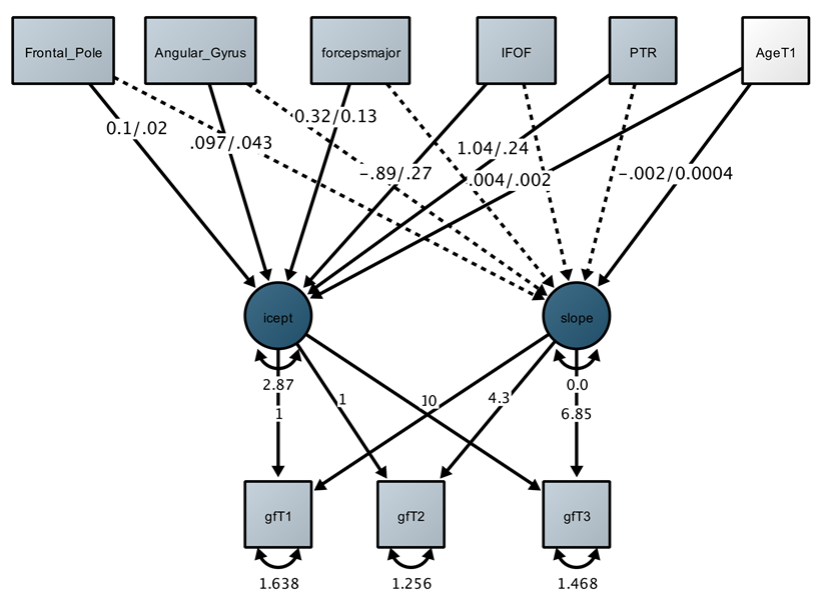
Final Multiple Indicator, Multiple Causes (MIMIC) Latent Growth (LGM) of fluid intelligence and neural determinants (grey and white matter). The strongest predictions are the frontal pole grey matter volume and the posterior thalamic radiations, both such that greater volume and greater Fractional Anisotropy (FA) were associated with better scores. All paths shown are beta/standard errors. Non-significant tracts and region covariances were estimated but are omitted for clarity.

## Discussion

### Summary of main findings

We conducted a preregistered examination of longitudinal changes in fluid intelligence in an N=185,317 subset of the Biobank cohort ( Sudlow *et al.*, 2015). We observed a negative effect of age on the fluid intelligence intercept, consistent with other cross-sectional studies, but smaller than normally found (cf. Kievit *et al.*, 2014). However, contrary to our expectations, our analysis of the rate of change of fluid intelligence revealed a positive rather than negative slope. In other words, rather than show decline, performance on the Biobank fluid intelligence task improved across test occasions, likely due to retest and practice effects. We also found a small negative effect of initial age on the rate of change, i.e. older people showed less improvement across time points. Convergence problems (likely due to the limited number of waves) meant that we were unable to infer whether the rates of change were best captured by a linear or quadratic model. No compelling evidence was observed for the existence of subgroups.

In a second set of analyses, we examined the neural determinants of individual differences in fluid intelligence. The absence of slope variance precluded meaningful modelling of individual differences in rate of change. In line with our expectations, we observed seven distinct and complementary contributions from individual white matter tracts. However, the effect sizes were small, and contrary to our expectations and previous work ( Kievit *et al.*, 2014; Kievit *et al.*, 2016), frontal white matter tracts were not among the strongest determinants of fluid abilities. The posterior thalamic radiations appeared as the strongest white matter predictor in both the white matter only model, as well as the combined grey matter/white matter model. The posterior thalamic radiations connect thalamic systems to both parietal and early visual systems. A tentative interpretation could be that parietal systems are often recruited in demanding tasks (e.g. Fedorenko *et al.*, 2013). However, the small magnitude of the effect size, as well as the relative dearth of previous findings relating the PTR to fluid reasoning (although some weak effects have been reported, e.g. Navas-Sánchez *et al.*, 2014), together suggest caution in interpreting this finding with confidence. Focusing on grey matter, we observed a strong, positive association between grey matter volume in the frontal pole and fluid intelligence, in line with our previous findings ( Kievit *et al.*, 2014), and two additional smaller positive effects of the angular gyri and the inferior frontal gyrus, together explaining (alongside age) 4.5% of the intercept variance in fluid intelligence. The relatively strong association of frontal pole grey matter volume is in line with our previous work in a healthy aging cohort ( Kievit *et al.*, 2014) as well as functional imaging findings (e.g., Kroger *et al.*, 2002) and lesion studies (e.g. Gläscher *et al.*, 2010). The relatively strong association of frontal pole grey matter volume is in line with our previous work in a healthy aging cohort ( Kievit *et al.*, 2014) as well as functional imaging findings (e.g., Kroger *et al.*, 2002) and lesion studies (e.g. Gläscher *et al.*, 2010). Finally, a joint model of grey and white matter revealed that both neural measures made unique contributions to fluid intelligence, supporting previous findings ( Kievit *et al.*, 2014) as well as our preregistered prediction (2c, pre-registration).

### Quality of the fluid intelligence measure

A plausible explanation for both the disparity in the size of cross-sectional age effects on fluid intelligence intercept (e.g. r=-0.04 in Figure 1, versus r=-0.55 in comparable samples), as well as the absence of expected slope effects, most likely lies in the fluid intelligence task itself. First and foremost, not all items are representative of classic fluid intelligence items. For instance, item two asks ‘which number is the largest?’. This item might be best characterized as relying on crystallized knowledge, and would not usually be considered a component of fluid intelligence. It would perhaps be more appropriate in a dementia-screening task in elderly samples than in a fluid intelligence test administered in a population-representative sample. This interpretation is supported by a striking ceiling effect on this item (99.06% accuracy). Similar ceiling effects were observed for other items (94.9% for the first item). However, other items (e.g. item 3) rely on verbal analogies, which likely do require a measure of abstract reasoning abilities. Taken together, individual differences in the mean (intercept) scores likely reflect fluid abilities to some degree, but more weakly so than traditional, standardized tests. Previous work on the Biobank fluid intelligence task has characterized the nature of the test as ‘verbal-numerical reasoning’ ( Lyall *et al.*, 2016), which is a more apt description than ‘fluid intelligence’, although arguably doesn’t cover items such as the example above. As for the longitudinal component, the relative memorability of certain items (such as the ‘largest number’ question) may help explain the absence of slope variance over time, as people are likely to provide the same answers on repeat testing occasions. Moreover, the self-paced nature of the task means that item 13 was only attempted by 4,350 out of 165,097 individuals at time point 1. Out of these participants, only 844 got the item correct, giving an overall accuracy rate of 0.5%. In short, the fluid intelligence task as currently implemented shows poor construct validity, and is vulnerable to ceiling and floor effects. Moreover, the self-paced nature (the total score reflects the number of correct items given within a 2-minute window) may exacerbate retest effects, given that remembering previous answers (right or wrong) and increased familiarity with the testing environment might lead to more items being attempted. Together, these properties may explain the absence of hypothesized longitudinal effects. Recently, Biobank has started acquiring a new fluid intelligence ‘matrix pattern completion’ task which more closely aligns with traditional psychometric tests of fluid intelligence. We expect that this novel subtest will show more robust age and neuroimaging effects.

## Conclusion

Many studies, particularly in neuroimaging, are underpowered ( Button *et al.*, 2013). The field’s effort to collect large, collaborative datasets is an important response to this scientific challenge. Biobank offers a uniquely rich, publicly-available dataset that has revolutionized the scope of large scale shared projects, and already led to numerous insights into the genetic, environmental and neural markers of healthy aging (e.g. Hagenaars *et al.*, 2016; Miller *et al.*, 2016; Muñoz *et al.*, 2016). However, our current analyses of the Biobank cognitive data demonstrate that the size of the dataset cannot always overcome suboptimal data quality ( Kolossa & Kopp, 2018). Longitudinal measurements may be especially vulnerable to practical constraints in large cohorts (e.g. short administration time, ease of use of the test etc.). Further improvements in the quality of cognitive data and additional waves of longitudinal measures will likely allow for more conclusive answers about the neural determinants of age-related changes in fluid intelligence, and facilitate understanding of lifespan changes in cognitive function.

## Data availability

Our analysis is based on data from the Biobank cohort, and as such cannot be attached in the raw form without violation contractual agreements. Our analyses can be reproduced (or improved) by the following three steps:

1) Create an account and enter a data access request through the Biobank portal, requesting the key variables in this manuscript (the fluid intelligence score, id 20016; the diffusion MRI tract averages for FA, id 134; and grey matter volume measures, id 110.

2) Run the script ‘Kievit_etal_biobank_dataprep.R’, provided in the supplementary materials ( Supplementary File 1). This will translate the biobank data object into an appropriately organized subset (‘Fulldat1.Rdata’ and ‘gfonly.dat’) ready for further processing.

3) Run the script Kievit_etal_biobank_analysis.R ( Supplementary File 2) on the data object (‘Fulldat1.R’) created using the ‘Kievit_etal_biobank_dataprep.R’ ( Supplementary File 1). This script will reproduce all analyses, as well as figures, reported in the above manuscript. The only exception is the growth mixture models – This can at present not be run in R. To this end, run the script Kievitetal_GFGMM1.inp ( Supplementary File 3) in Mplus, modifying the line ‘CLASSES = c (1);’ to vary the number of latent classes.

## Notes

^1^Note: To achieve stable model estimation convergence we had to switch the estimator from MLR to ML.

## Supplementary material

Files allowing the replication of the presented analysis

Supplementary File 1 - Kievit_etal_biobank_dataprep.R

Supplementary File 2 – Kievit_etal_biobank_analysis.R

Supplementary File 3 – Kievitetal_GFGMM1.inp
