# Supplementary material for: The neural determinants of age-related changes in fluid intelligence: a pre-registered, longitudinal analysis in UK Biobank
Source: Wellcome Open Res. 2018 Jun 15;3:38. Originally published 2018 Apr 5. [Version 2] doi: 10.12688/wellcomeopenres.14241.2 (PMC5909055; doi:10.12688/wellcomeopenres.14241.2)
Supplement: Peer review history

# Peer review: Referee response for version 2

**DOI:** 10.21956/wellcomeopenres.15944.r33328

**Published:** 2018-06-29

**Authors:** Florian Schmiedek

**Affiliations:** 1 German Institute for International Educational Research (DIPF), Frankfurt, Germany

My earlier concerns and comments have been thoroughly addressed in this revised version. Changes made are all appropriate and explanations provided for not implementing some of the suggestions are clear and well comprehensible. No further queries from my side.

I have read this submission. I believe that I have an appropriate level of expertise to confirm that it is of an acceptable scientific standard.

# Peer review: Referee response for version 1

**DOI:** 10.21956/wellcomeopenres.15497.r32843

**Published:** 2018-05-04

**Authors:** Florian Schmiedek

**Affiliations:** 1 German Institute for International Educational Research (DIPF), Frankfurt, Germany

This is a well-conducted set of preregistered analyses, addressing important research questions, and using an impressive longitudinal data set. While the chosen latent growth model generally is standard for analyzing questions pertaining average longitudinal change (and individual differences therein) with few measurement occasions, there are some aspects that should or could be done somewhat differently, or additionally, in my view.

 1. I would refrain from attempts to model quadratic change with (at maximum) only three measurement occasions. As this was part of the pre-registration, it should be mentioned, but maybe together with a qualification that a quadratic change model is not generally identified with 3 time points and identification could only be achieved using constraints that were not specified a priori (e.g., constraining the linear slope variance to zero).

 2. Similarly, I find the use of a “boost” factor problematic, as the implied assumption of retest effects only taking place between T1 and T2 is difficult to defend. Also, this model has the same identification problem as a quadratic model.

 3. Power to detect individual differences in change may be larger if individual differences in the true timing of the measurement occasions would be taken into account in the models (instead of using the mean intervals). In an SEM framework, this is possible using Mplus and the TSCORES option. I would encourage trying out this modeling option and would consider it a minor deviation from the pre-registration, as it would keep with the general modeling strategy and just mean using all available information to get most precise and powerful parameter estimates.

 4. In an exploratory manner, I would encourage to pursue the attempt to use measurement models for the fluid intelligence construct a bit more and reduce the set of items to those that “are representative of classic fluid intelligence items”. This may improve model fit and help model convergence, and may also increase power to detect variance in slopes. Based on a decent and time-invariant measurement model, latent change score models could also allow to model change from T1 to T2 and change from T2 to T3 separately (capturing potential quadratic change or differential retest effects). Related to this point, I would like to see a brief but complete description of all fluid intelligence items in the Methods section.

 5. As the power to detect significant variance in slopes and the power to detect effects of certain moderator variables on the slope may differ, I do not think that it is precluded to test such moderation effects just because the variance in slopes turns out not to be significant. As the moderator effects pertain to pre-registered a-priori hypotheses, I would go ahead and test and report these effects (preferably using likelihood ratio tests based on model comparisons), even though the variance of slopes may not be significant.

 6. Generally, the variance of the slope factor should not be evaluated with z-test, but also with likelihood ratio tests, using adjusted critical values (see Stoel et al., 2006 ^1^). Implicitly, this is already done by reporting the chi2 values for models with and without the slope variance. I would fully replace the reported z and p values with the more appropriate LR test, however.

 Minor points

 The term “slope intercept” may be confusing. Maybe use “the regression intercept of a model with the slope factor as dependent variable” or some other explanation that helps to distinguish between “intercept” as growth factor and “intercept” as regression parameter in the MIMIC models.

I have read this submission. I believe that I have an appropriate level of expertise to confirm that it is of an acceptable scientific standard, however I have significant reservations, as outlined above.

## References

[1] StoelRDGarreFGDolanCvan den WittenboerG: On the likelihood ratio test in structural equation modeling when parameters are subject to boundary constraints. *Psychol Methods*.2006;11(4) : 10.1037/1082-989X.11.4.439 439-55 10.1037/1082-989X.11.4.439 17154756

# Response

**Published:** 2018-05-25

**Authors:** Rogier Kievit

**Affiliations:** Dr, UK

'This is a well-conducted set of preregistered analyses, addressing important research questions, and using an impressive longitudinal data set. While the chosen latent growth model generally is standard for analyzing questions pertaining average longitudinal change (and individual differences therein) with few measurement occasions, there are some aspects that should or could be done somewhat differently, or additionally, in my view.'

*We thank the reviewer for their comments, which have served to strengthen the paper*

1. I would refrain from attempts to model quadratic change with (at maximum) only three measurement occasions. As this was part of the pre-registration, it should be mentioned, but maybe together with a qualification that a quadratic change model is not generally identified with 3 time points and identification could only be achieved using constraints that were not specified a priori (e.g., constraining the linear slope variance to zero).

*We agree that we should have mentioned the necessity of constraints in the pre-registration. We had hoped to be able to model decline as a function of age rather than testing occasion which would have allowed more flexibility in this regard. We did mention this in passing in the original manuscript as follows, **‘and imposed constraints in order to render the model identifiable (residual variances equality constrained across occasions, and linear slope variance constrained to 0 based on the linear model).’** We now also include an explication of the need of constraints below in the boost factor*

2. Similarly, I find the use of a “boost” factor problematic, as the implied assumption of retest effects only taking place between T1 and T2 is difficult to defend. Also, this model has the same identification problem as a quadratic model.

*We agree that we should have spelled out the challenges with estimating more an additional growth factor with so few timepoints more clearly in the pre-registration, and have now explained these limitations in the revision. Conceptually, we find the boost factor intuitively plausible – The change between wave 1 and 2 would entail the familiarity with the setting, using the iPad, the time constraints etcetera. This would be much less strong between wave 2 and 3. This type of boost factor was included as a core retest mechanisms in a simulation based paper on modelling retest effects (Hoffman, L., Hofer, S. M., & Sliwinski, M. J, 2011) and was shown to have a less negative effect on the estimation on other model parameters than incremental practice effects.*

**This so-called ‘boost’ factor (Hoffman, Hofer, & Sliwinski, 2012) captures the hypothesis that test performance will show an improvement between the first and second testing occasions that is purely a practice effect. The inclusion of the boost factor rendered the slope intercept non-significant, which is compatible with the notion that the gains are most likely practice gains. However, like the quadratic model, such a more complex model is only identified by imposing a range of constraints (here including constraining the boost factor variance to 0). Moreover, despite these constraints this model yielded an improper solution and should thus be interpreted with caution.**

3. Power to detect individual differences in change may be larger if individual differences in the true timing of the measurement occasions would be taken into account in the models (instead of using the mean intervals). In an SEM framework, this is possible using Mplus and the TSCORES option. I would encourage trying out this modeling option and would consider it a minor deviation from the pre-registration, as it would keep with the general modeling strategy and just mean using all available information to get most precise and powerful parameter estimates.

*We agree this is a principled and elegant manner to model these effects. However, despite increasing the EM iterations well beyond the Mplus default, this model did not converge. Nonetheless we agree it is the more principled choice so have now included it in the manuscript as follows:*

**Here we use the mean age interval between waves to guide the fixed factor loadings in the growth model. A more precise modelling approach is to use the individual ages at each timepoint. This is known as a ‘definition variable’ approach (Mehta & Neale), uses all the information present in the data in richer manner and can be implemented in either Mplus or OpenMx (but not yet Lavaan). However, in the present dataset this approach did not converge**

4. In an exploratory manner, I would encourage to pursue the attempt to use measurement models for the fluid intelligence construct a bit more and reduce the set of items to those that “are representative of classic fluid intelligence items”. This may improve model fit and help model convergence, and may also increase power to detect variance in slopes.

*In our previous manuscript we fit a full second order latent growth curve model which would allow individual items to contribute more, or less, to the latent factor. As reported this did not yield acceptable model fit nor meaningfully changed our findings. Moreover, we have now refit the models with each individual item, again yielding virtually identical results. In short we believe no meaningful signal can be extracted from these items without exhaustive data-driven subselection that may lead to overfitting.*

**

**In a final exploratory analysis, we reran the basic growth model with every individual item. This yielded qualitatively very similar results, with positive slopes for all items and non-significant slopes for all but one item (item 5). Closer inspection of item 5 suggested only a marginal, uncorrected benefit of freely estimating the slope variance χ ^2^(1), 8.1, p=.004, combined with a non-significant slope intercept, and a BIC favouring the constrained slope model, together suggesting insufficient evidence to proceed with this post hoc item selection instead of the sumscore.**

Based on a decent and time-invariant measurement model, latent change score models could also allow to model change from T1 to T2 and change from T2 to T3 separately (capturing potential quadratic change or differential retest effects).

*We agree in principle, but in practice these desiderata of the model fit and item properties seem beyond the data quality present in Biobank.*

Related to this point, I would like to see a brief but complete description of all fluid intelligence items in the Methods section.

*We included a link to the complete set of questionnaire items which is available online here:*

*https://biobank.ctsu.ox.ac.uk/crystal/docs/Fluidintelligence.pdf*

*We have modified the wording in the manuscript to be more explicit (as it currently only states ‘the manual’)*

**for a complete overview of the 13 individual fluid intelligence items, please see http://biobank.ctsu.ox.ac.uk/crystal/docs/Fluidintelligence.pdf**

5. As the power to detect significant variance in slopes and the power to detect effects of certain moderator variables on the slope may differ, I do not think that it is precluded to test such moderation effects just because the variance in slopes turns out not to be significant. As the moderator effects pertain to pre-registered a-priori hypotheses, I would go ahead and test and report these effects (preferably using likelihood ratio tests based on model comparisons), even though the variance of slopes may not be significant.

*We agree that the significance of the slope in isolation needn’t be a guiding principle to guide the analysis of moderators. Note that all continuous predictors of slope variance in our models are still included even in the models where slope (residual) variance is constrained to 0 – in other words, all continuous neural moderators of slope were included, but proved non-significant. We now include an LRT for each relevant model, comparing one where all neural predictors of slope are freely estimated versus a model where they are constrained to 0 for white matter, grey matter and the combined model. In all case the constrained model is preferred.*

**None of the white tracts predicted slope variance - A likelihood ratio test showed that the regression paths of the slope on the individual tracts could be constrained to 0 without adversely affecting model fit χ ^2^ (15), 17.97, p=.26.**

****

**No regions predicted slope variance - A likelihood ratio test showed the regression paths of the slope on the individual regions could be constrained to 0 without adversely affecting model fit χ ^2^ (10), 12.55, p=.24.**

****

**As before, no regions or tracts predicted slope variance, χ ^2 ^(10), 10.99, p=.35.**

6. Generally, the variance of the slope factor should not be evaluated with z-test, but also with likelihood ratio tests, using adjusted critical values (see Stoel et al., 2006 ^1^). Implicitly, this is already done by reporting the chi2 values for models with and without the slope variance. I would fully replace the reported z and p values with the more appropriate LR test, however.

*We agree entirely. We reported the Wald test for reasons of greater familiarity to most readers, as well as slightly fewer issues of model convergence due to variance constraints, but on reflection we agree that the LR test is more appropriate and have updated this for all variance parameters.*

Minor points

The term “slope intercept” may be confusing. Maybe use “the regression intercept of a model with the slope factor as dependent variable” or some other explanation that helps to distinguish between “intercept” as growth factor and “intercept” as regression parameter in the MIMIC models.

*We agree ‘slope intercept’ can be confusing. We have clarified the first mention of this term in parentheses*

**First, the slope intercept (in this specification, the mean change per measurement occasion)**

# Peer review: Referee response for version 1

**DOI:** 10.21956/wellcomeopenres.15497.r32845

**Published:** 2018-04-19

**Authors:** Sara Pudas, Michael Rönnlund

**Affiliations:** 1 Department of Psychology, Umeå University, Umeå, Sweden; 2 Umeå Center for Functional Brain Imaging, Umeå University, Umeå, Sweden

The study involves a pre-registered analysis, is hypothesis-driven, and seems to involve sound analyses of the data and the text is clear. Nevertheless, we have a couple of concerns in regard to the presentation and data analyses.

 Introduction: The authors state that “Both cross-sectional and longitudinal studies have shown that advancing age is associated with a marked decrease in fluid intelligence starting in the third or fourth decade of life”, citing work by Hartshorne and Germine (2015) ^1^ and Schaie (1994) ^2^. However, longitudinal data in the latter article clearly indicate a higher age of onset of mean-level decline than this, for each of the Primary Mental Abilities (around age 60; see Figure 2), including Inductive reasoning, a core facet of fluid intelligence (narrowly defined). The study by Hartshorne and Germaine involved cross-sectional data. Thus, whereas cross-sectional data typically indicate decline in the third or fourth decade of life (or earlier, see Park et al., 2002 ^3^) actual decline at the mean level may appear later, at least as judged by the data in Schaie (1994). This is relevant to note as, from that perspective, quite a few participants in the UK biobank study (range 39-73 years) might be expected to be rather stationary in regard to mean-level fluid intelligence over a relatively short test-retest interval.

 Results: Regarding attrition, did the participants who participated in 2 or 3 test waves differ from those who dropped out after the first occasion? Describing drop-out mechanisms with regards to age, gender, fluid intelligence at baseline, and potentially socio-economic factors (if such are available) may help to clarify why the average slope was positive.

 Discussion: It would be informative if the authors could comment on the validity of their findings regarding gray and white matter predictors of level of fluid intelligence. Despite challenges with the task validity and psychometric properties, are the significant relationships that were observed plausible (albeit smaller in magnitude than expected)? For instance, is it reasonable that the posterior thalamic radiations had the strongest association with fluid intelligence (despite contradicting the authors own previous work)? Were the relative contributions of gray and white matter variables in line with previous literature?

 The direction of the effect between two of the white matter tracts and fluid intelligence appears opposite to expectations (the forceps major and the inferior fronto-occipital fasciculus).

**Minor comments:**

 Methods section: Please specify if the 27 white matter tracts were the total number of tracts available for this data set.

 Methods section: please state whether the gray matter volumes were raw values or corrected for intracranial volume (which could have made sense given the aging-related original hypotheses)?

 P. 8, the last sentence states that no regions explained significant variance in slope. But wasn’t slope variance constrained to be zero in this model?

We have read this submission. We believe that we have an appropriate level of expertise to confirm that it is of an acceptable scientific standard.

## References

[1] HartshorneJKGermineLT: When does cognitive functioning peak? The asynchronous rise and fall of different cognitive abilities across the life span. *Psychol Sci*.2015;26(4) : 10.1177/0956797614567339 433-43 10.1177/0956797614567339 25770099PMC4441622

[2] SchaieKW: The course of adult intellectual development. *Am Psychol*.1994;49(4) :304-13 820380210.1037//0003-066x.49.4.304

[3] ParkDCLautenschlagerGHeddenTDavidsonNSSmithADSmithPK: Models of visuospatial and verbal memory across the adult life span. *Psychol Aging*.2002;17(2) :299-320 12061414

# Response

**Published:** 2018-05-25

**Authors:** Rogier Kievit

**Affiliations:** Dr, UK

The study involves a pre-registered analysis, is hypothesis-driven, and seems to involve sound analyses of the data and the text is clear. Nevertheless, we have a couple of concerns in regard to the presentation and data analyses.

*We thank the reviewers for their comments, which have served to strengthen the paper*

 Introduction: The authors state that “Both cross-sectional and longitudinal studies have shown that advancing age is associated with a marked decrease in fluid intelligence starting in the third or fourth decade of life”, citing work by Hartshorne and Germine (2015) ^1^ and Schaie (1994) ^2^. However, longitudinal data in the latter article clearly indicate a higher age of onset of mean-level decline than this, for each of the Primary Mental Abilities (around age 60; see Figure 2), including Inductive reasoning, a core facet of fluid intelligence (narrowly defined). The study by Hartshorne and Germaine involved cross-sectional data. Thus, whereas cross-sectional data typically indicate decline in the third or fourth decade of life (or earlier, see Park et al., 2002 ^3^) actual decline at the mean level may appear later, at least as judged by the data in Schaie (1994). This is relevant to note as, from that perspective, quite a few participants in the UK biobank study (range 39-73 years) might be expected to be rather stationary in regard to mean-level fluid intelligence over a relatively short test-retest interval.

*We agree that we oversimplified the state of knowledge, and did not use the optimal references to support our claim. However, it is also likely also the case that longitudinal data might underestimate within-subject decline to some degree due to retest or practice effects (e.g. Salthouse, T. A., Schroeder, D. H., & Ferrer, E. (2004), with Salthouse (2009) estimating decline to begin in the third or fourth decade. Regardless of the precise decade, we would suggest that one would not expect a slope increase or a non-significant slope variance in a sample of this age range.  The rephrased section reads as follows:*

**Both cross-sectional (e.g. Hartshorne & Germine, 2015; Kievit et al., 2016) and longitudinal studies (e.g. Salthouse, 2009, Schaie, 1994; Ghisletta et al., 2012) have shown that advancing age is associated with a marked decrease in fluid intelligence performance. Although the precise starting point of decline is hard to estimate precisely due to cohort effects, selective attrition and enrolment and retest effects in longitudinal cohorts (e.g. Salthouse, Schroeder & Ferrer, 2004), estimates for the onset of decline in fluid intelligence range between the third (e.g. Salthouse, 2009; Park et al., 2002) and sixth decade of life (e.g. Schaie, 1994).**

 Results: Regarding attrition, did the participants who participated in 2 or 3 test waves differ from those who dropped out after the first occasion? Describing drop-out mechanisms with regards to age, gender, fluid intelligence at baseline, and potentially socio-economic factors (if such are available) may help to clarify why the average slope was positive.

*These participants differed slightly – those who participated in all three waves were about 6 months older on average, and had slightly higher fluid intelligence scores at T1 (see new plots in Figure 2). However, to the extent that these characteristics explain attrition (i.e. Missing At Random), our approach of full information maximum likelihood should adjust appropriately. This is confirmed by the highly similar results (non-significant slope variance, marginally positive slope) when we run the model only in those individuals who have data in all three waves. The conjunction of the results from the boost model, the task characteristics, the slight negative effect of age on slope and previous work on retest effects in longitudinal aging together strongly suggest the driving force behind the positive slope are small but significant retest effects due to increased familiarity with the task and setting, rather than a more substantively meaningful signal. We now include the below paragraphs as well as two new figures*

**Participants who took part in all three waves (N=870) were slightly older, and had lightly higher baseline scores, than those who took part in only one or two waves (See Figure 2A and 2B) – A common pattern of selective attrition. By using all available data, under the assumption of Missing At Random (i.e. the attrition is associated with variables also included in the model) using Full Information Maximum likelihood should yield unbiased estimates (cf.  Enders & Bandalos, 2001).**

 Discussion: It would be informative if the authors could comment on the validity of their findings regarding gray and white matter predictors of level of fluid intelligence. Despite challenges with the task validity and psychometric properties, are the significant relationships that were observed plausible (albeit smaller in magnitude than expected)? For instance, is it reasonable that the posterior thalamic radiations had the strongest association with fluid intelligence (despite contradicting the authors own previous work)? Were the relative contributions of gray and white matter variables in line with previous literature?

*The frontal pole grey matter finding is in line with previous findings from our lab as well as others – we have now clarified this as follows*

**The relatively strong association of frontal pole grey matter volume is in line with our previous work in a healthy aging cohort (Kievit et al. 2014) as well as functional imaging findings (e.g., Kroger et al., 2002) and lesion studies (e.g. Gläscher et al., 2010).**

*The absence of Forceps Minor as a strong predictor and the presence of PTR as a predictor are contrary to previous findings. We now discuss tentatively as follows:*

**The posterior thalamic radiations appeared as the strongest white matter predictor in both the white matter only model, as well as the combined grey matter/white matter model. The posterior thalamic radiations connect thalamic systems to both parietal and early visual systems. A tentative interpretation could be that parietal systems are often recruited in demanding tasks (e.g. Fedorenko et al, 2013). However, the small magnitude of the effect size, as well as the relative dearth of previous findings relating the PTR to fluid reasoning (although some weak effects have been reported, e.g. Navas‐Sánchez et al. 2014), together suggest caution in interpreting this finding with confidence.**

 The direction of the effect between two of the white matter tracts and fluid intelligence appears opposite to expectations (the forceps major and the inferior fronto-occipital fasciculus).

*Indeed, this is (weakly) opposite to our and other previous findings. However, refitting the model with only each of these individual tracts (and age) removes this negative ‘effect’, so we suspect the weakly negative effects are a consequence of collinear predictors in a very large sample, rather than paths to be interpreted strongly.­*

 Methods section: Please specify if the 27 white matter tracts were the total number of tracts available for this data set.

*This was indeed the full number of tracts available – We have clarified this in the manuscript*

**‘We included all 27 white matter tracts in the Biobank (Miller et al., 2016),’**

 Methods section: please state whether the gray matter volumes were raw values or corrected for intracranial volume (which could have made sense given the aging-related original hypotheses)?

                

*These were the raw values. Given various lines of evidence that suggest that larger overall brain volume is associated with intelligence (e.g. Gignac & Bates, 2017), we did not want to adjust in this manner (this is consistent with our previous approach, e.g. Kievit et al., 2014).  Various lines of evidence suggest that total brain volume change may be a leading indicator of declines in cognitive performance (e.g. Grimm, K. J., An, Y., McArdle, J. J., Zonderman, A. B., & Resnick, S. M. (2012) which would suggest actual grey matter volume is a highly relevant measure in aging populations.*

 P. 8, the last sentence states that no regions explained significant variance in slope. But wasn’t slope variance constrained to be zero in this model?

*Perhaps counterintuitively, constraining the slope variance effectively constrains the **residual, or conditional, **variance to 0, not the absolute variance– In other words, any predictors may still exert influence and be estimated as normal (although the standardized effect sizes will be artificially high). For instance, age significantly (but weakly) predicts the positive slope with identical parameter estimates regardless of the slope constraint (this is so in Mplus and Lavaan). An alternative, defensible approach would be to constrain all predictors of the slope to 0 whenever the slope variance is constrained. However, as this would gain a large number of degrees of freedom, thereby (arguably) artificially improving model fit based on purely data driven considerations, we chose against doing so.*

# Peer review: Referee response for version 1

**DOI:** 10.21956/wellcomeopenres.15497.r32839

**Published:** 2018-04-19

**Authors:** Donald M. Lyall

**Affiliations:** 1 Institute of Health and Wellbeing, University of Glasgow, Glasgow, UK

This paper aimed to investigate the neural substrates of fluid intelligence, and its change across time, using participants with cognitive and brain magnetic resonance imaging (MRI) data in UK Biobank.  The paper is thorough and well-written, and validly attempts to progress our understanding of this area of research. The authors found separate grey and white matter contributions to mean cognitive scores, however a major limitation related to the ‘fluid reasoning’ task itself, and its construct validity.

  

1. **Is the work clearly and accurately presented and does it cite the current literature?**
  Yes.
Minor suggestions
  You may be interested in our 2016 PLOS ONE paper ^1^ where we highlighted many of the same issues discussed here with the fluid reasoning task, although at that point not including any of the participants who had completed it at MRI. We suggest an alternative title for the task – ‘verbal-numerical reasoning’ (which you may or may not agree with).
  See Cox et al. ^2^  where in n=3,513 UK Biobank participants it is suggested that 1) five specific white matter tracts are perhaps better off not included in a single FA factor - namely middle cerebellar peduncle, bilateral medial lemniscus and parahippocampal cingulum - because these had low factor loadings, 2) additional tract integrity metrics (e.g. NODDI; MD) could be informative beyond FA, and 3) there were some left vs. right hemispheric differences in FA with age, contrasting with here where values were averaged across left and right hemispheres.
  Regarding the line: “We started with 27 tracts (Miller et al., 2016), and averaged bilateral hemispheric tracts, yielding mean FA estimates for a total of 15 tracts”, please elaborate on why you took this approach, vs. including more tracts.  
2. **Is the study design appropriate and is the work technically sound?**
  Yes.
Minor suggestions
  In the section regarding white/grey-matter contributions to fluid intelligence, have you considered looking specifically at contributions to scores in participants performing it for the first time at MRI?   
  Did you consider people who may have developed neurological/neurodegenerative conditions across waves?
  Please give more details on image quality control procedures – e.g. whether you performed anything beyond what UK Biobank have done centrally, and detail slightly more what UK Biobank have done.
3. **Are sufficient details of methods and analysis provided to allow replication by others?**
  Yes. Although it is worth noting that the UK Biobank is such that firstly the number of participant scans is increasing in batches (see http://www.fmrib.ox.ac.uk/ukbiobank/), and secondly that there are sometimes participant withdrawals from the sample – so researchers who downloaded data tomorrow and ran the script might not find precisely the same results.
4. **If applicable, is the statistical analysis and its interpretation appropriate?**
  Yes.
Minor suggestions
  The authors refer to the ‘fluid intelligence’ task including some ‘crystal-type’ items (e.g. ‘which number is the largest’). Have you considered dropping these items from the total score? Scores on the individual items are available.  
5. **Are all the source data underlying the results available to ensure full reproducibility?**
  Yes. Data application must be approved and sought by researchers from UK Biobank ( http://www.ukbiobank.ac.uk/). The authors note this, and provide good directives regarding procuring the relevant data. Scripts and notes are provided.
6. **Are the conclusions drawn adequately supported by the results?**
  Yes. I agree that a key limitation is the ‘poor construct validity’ of the fluid intelligence measure.

I have read this submission. I believe that I have an appropriate level of expertise to confirm that it is of an acceptable scientific standard.

## References

[1] LyallDCullenBAllerhandMSmithDMackayDEvansJAndersonJFawns-RitchieCMcIntoshADearyIPellJ: Cognitive Test Scores in UK Biobank: Data Reduction in 480,416 Participants and Longitudinal Stability in 20,346 Participants. *PLOS ONE*.2016;11(4) : 10.1371/journal.pone.0154222 10.1371/journal.pone.0154222 PMC484416827110937

[2] CoxSRitchieSTucker-DrobELiewaldDHagenaarsSDaviesGWardlawJGaleCBastinMDearyI: Ageing and brain white matter structure in 3,513 UK Biobank participants. *Nature Communications*.2016;7: 10.1038/ncomms13629 10.1038/ncomms13629 PMC517238527976682

# Response

**Published:** 2018-05-25

**Authors:** Rogier Kievit

**Affiliations:** Dr, UK

1.  You may be interested in our 2016 PLOS ONE paper ^1^ where we highlighted many of the same issues discussed here with the fluid reasoning task, although at that point not including any of the participants who had completed it at MRI. We suggest an alternative title for the task – ‘verbal-numerical reasoning’ (which you may or may not agree with).

*We agree this is a highly pertinent paper and now refer to it more explicitly. Although verbal-numerical reasoning is closer to the nature of the task, in our view, certain items do not require any reasoning at all (e.g. ‘which is the largest number’), so although closer it doesn’t cover all items. To avoid confusion we therefore maintained the Biobank nomenclature.*

**Previous work on the Biobank fluid intelligence task has characterized the nature of the test as ‘verbal-numerical reasoning’ (Lyall et al., 2016), which is a more apt description than ‘fluid intelligence’, although arguably doesn’t cover items such as the example above.**

 See Cox et al. ^2^  where in n=3,513 UK Biobank participants it is suggested that 1) five specific white matter tracts are perhaps better off not included in a single FA factor - namely middle cerebellar peduncle, bilateral medial lemniscus and parahippocampal cingulum - because these had low factor loadings, 2) additional tract integrity metrics (e.g. NODDI; MD) could be informative beyond FA, and 3) there were some left vs. right hemispheric differences in FA with age, contrasting with here where values were averaged across left and right hemispheres.

*1) In our view the global factor is a particular hypothesis, namely whether white matter integrity can be adequately captured by a single factor – In that sense, removing tracts would be a suboptimal way to answer that question. Of course if the purpose is to capture a large amount of the variance across tracts in a single summary measure then something like a PCA (e.g. Penke et al., 2012) would be appropriate, but here we wanted to specifically test whether a single factor could adequately summarize the tract covariance, and found it did not (note that one could argue the model fit of especially other metrics such as MD in the above paper below common cut offs, so even with these tracts removed one could argue there is evidence of specificity beyond the global factor).*

*2) We agree these metrics may be of interest for future approaches, and mention these other metrics more explicitly as possibilities in the discussion. As FA aligns with our previous cohorts (and other analyses) that inspired our pre-registered hypotheses we stuck with FA for the actual analyses. We clarify as follows*

**Note that Biobank also includes various other white matter metrics of interest including diffusivity (MD), Neurite Orientation and Dispersion and others – These measures have specific strengths and weaknesses (see Cox et al., 2016, for a discussion of the merits of more novel metrics) that are beyond the remit of this manuscript.**

 3) Regarding the line: “We started with 27 tracts (Miller et al., 2016), and averaged bilateral hemispheric tracts, yielding mean FA estimates for a total of 15 tracts”, please elaborate on why you took this approach, vs. including more tracts.  

*27 tracts are all the tracts in Biobank.  We agree our phrasing was imprecise and could be read as suggesting a sub selection of even more tracts than 27, so we have adjusted our phrasing accordingly (another reviewer had the same query). We had no specific hypotheses regarding lateralization, and two pragmatic considerations in favour of bilateral averaging.  First, inclusion of the individual tracts considerably increases the size of the covariance matrix, which can complicate estimation. Second, simultaneous inclusion of highly collinear predictors in e.g. a MIMIC model can lead to estimation problems. Moreover, in the case of highly collinear predictors, this can artificially increase the difference between the predictors (with one tract highly significant, the other non-significant) merely because they have very similar predictions.*

**

 In the section regarding white/grey-matter contributions to fluid intelligence, have you considered looking specifically at contributions to scores in participants performing it for the first time at MRI?   

*The prediction of the intercept score for each individual will be extremely similar to the current model which predicts fluid intelligence score intercepts.*

 Did you consider people who may have developed neurological/neurodegenerative conditions across waves?

*We did not consider this – to the extent that a subset of participants in a cohort of this magnitude will inevitably display pre-clinical symptoms we consider that part a natural variation in a large sample and should therefore be captured. Moreover, our attempt at fitting a growth mixture model did not yield a clearly identifiable subgroup of individuals with relative rapidly decline, above and beyond what would be expected as a function of a normal population distribution of slopes.*

**

 Please give more details on image quality control procedures – e.g. whether you performed anything beyond what UK Biobank have done centrally, and detail slightly more what UK Biobank have done.

*We have expanded this section as follows:*

**We started with 27 tracts averages as generated by Biobank**

****

 and

****

**Quality control was conducted by both automated identification of e.g. outlier slices and SNR, as well as manual inspection – For more detail, see Miller et al. 2016, online methods.**

  The authors refer to the ‘fluid intelligence’ task including some ‘crystal-type’ items (e.g. ‘which number is the largest’). Have you considered dropping these items from the total score? Scores on the individual items are available.

*We have now included the below*

**In a final exploratory analysis, we reran the basic growth model with every individual item. This yielded qualitatively very similar results, with positive (but largely non-significant) slopes for all items and non-significant slope variances for all but one item (item 5). Closer inspection of item 5 suggested only a marginal, uncorrected benefit of freely estimating the slope variance χ ^2^(1), 8.1, p=.004, combined with a non-significant slope intercept, and a BIC favouring the constrained-to-0 slope model, together suggesting insufficient evidence to proceed with this post hoc item selection instead of the sumscore.**
